# CrossDome: an interactive R package to predict cross-reactivity risk using immunopeptidomics databases

**DOI:** 10.3389/fimmu.2023.1142573

**Published:** 2023-06-12

**Authors:** Andre F. Fonseca, Dinler A. Antunes

**Affiliations:** Antunes Lab, Center for Nuclear Receptors and Cell Signaling (CNRCS), Department of Biology and Biochemistry, University of Houston, Houston, TX, United States

**Keywords:** T-cell cross-reactivity prediction, off-target toxicity, antigen prioritization, T-cell therapy, MAGEA3, cancer immunotherapy, computational oncology

## Abstract

T-cell-based immunotherapies hold tremendous potential in the fight against cancer, thanks to their capacity to specifically targeting diseased cells. Nevertheless, this potential has been tempered with safety concerns regarding the possible recognition of unknown off-targets displayed by healthy cells. In a notorious example, engineered T-cells specific to MAGEA3 (EVDPIGHLY) also recognized a TITIN-derived peptide (ESDPIVAQY) expressed by cardiac cells, inducing lethal damage in melanoma patients. Such off-target toxicity has been related to T-cell cross-reactivity induced by molecular mimicry. In this context, there is growing interest in developing the means to avoid off-target toxicity, and to provide safer immunotherapy products. To this end, we present CrossDome, a multi-omics suite to predict the off-target toxicity risk of T-cell-based immunotherapies. Our suite provides two alternative protocols, i) a peptide-centered prediction, or ii) a TCR-centered prediction. As proof-of-principle, we evaluate our approach using 16 well-known cross-reactivity cases involving cancer-associated antigens. With CrossDome, the TITIN-derived peptide was predicted at the 99+ percentile rank among 36,000 scored candidates (p-value < 0.001). In addition, off-targets for all the 16 known cases were predicted within the top ranges of relatedness score on a Monte Carlo simulation with over 5 million putative peptide pairs, allowing us to determine a cut-off p-value for off-target toxicity risk. We also implemented a penalty system based on TCR hotspots, named contact map (CM). This TCR-centered approach improved upon the peptide-centered prediction on the MAGEA3-TITIN screening (e.g., from 27th to 6th, out of 36,000 ranked peptides). Next, we used an extended dataset of experimentally-determined cross-reactive peptides to evaluate alternative CrossDome protocols. The level of enrichment of validated cases among top 50 best-scored peptides was 63% for the peptide-centered protocol, and up to 82% for the TCR-centered protocol. Finally, we performed functional characterization of top ranking candidates, by integrating expression data, HLA binding, and immunogenicity predictions. CrossDome was designed as an R package for easy integration with antigen discovery pipelines, and an interactive web interface for users without coding experience. CrossDome is under active development, and it is available at https://github.com/AntunesLab/crossdome.

## 1 Introduction

T-cell-based therapies are providing promising approaches for treating several types of cancer. These therapies leverage the cellular immunity mechanisms allowing T-cell lymphocytes to surveil, recognize, and eliminate cells displaying at their surfaces “foreign” peptides bound to Human Leukocyte Antigen (HLA) receptors (1, 2). This class of cancer immunotherapy treatments include i) the use of peptide-based vaccines to trigger the expansion of tumor-specific T-cells (3–6), and ii) the use of adoptive T-cell therapy, which involves collecting, expanding, and transferring tumor-specific T-cells to treat cancer patients (7, 8). In this context, the therapeutic T-cells can be unaltered tumor infiltrating lymphocytes, or genetically modified T-cells engineered to have higher affinity against specific tumor-associated antigens (4, 9, 10). Recently, more effective control of cancer progression was achieved with combined use of adoptive T-cell therapy and immune checkpoint inhibitors (11, 12). There is also growing interest in the development of chimeric antigen receptor T-cells (CAR-T), and tumor-specific antibodies that mimic T-cell receptor (TCR) recognition (13, 14).

However, several limitations are still hindering the broader use of T-cell therapies for cancer treatment (15). For instance, engineering a T-cell receptor is a challenging task that involves potentially conflicting goals, such as enhancing the T-cell response to the tumor-derived peptide, while avoiding side effects caused by T-cell cross-reactivity (16–18). T-cell cross-reactivity is the ability of a single T-cell clonotype to recognize and respond to multiple heterologous peptide-HLA (pHLA) complexes (19–21). From an evolutionary perspective, T-cell cross-reactivity is necessary to maximize the range of unrelated antigens/pathogens that can be recognized by a limited pool of T-cells (i.e., to mediate heterologous immunity between pathogens) (22–24). On the other hand, in T-cell-based therapy, cross-reactivity events have been linked to off-target toxicity risk, i.e., recognition of self-derived peptide-targets leading to autoimmune reactions against healthy tissues (21, 25).

To date, multiple clinical trials have been withdrawn due to T-cell cross-reactivity issues (26, 27). In the most notable example, MAGEA3-specific engineered T-cells were associated with severe off-target toxicity in melanoma patients. It was observed that these therapeutic T-cells were cross-reactive with a TITIN-derived peptide, causing lethal cardiotoxicity in at least five patients (26). Other cross-reactivity events have been reported in studies involving different tumor-associated antigens, such as MART-1, NY-ESO-1, and AFP (28–30). Additionally, off-target toxicity has also been reported with the use of CAR-T therapy (31–33). It is also important to note that while TCR engineering can increase the risk of dangerous T-cell cross-reactivities (34), this risk exists with any T-cell-based therapy, including the use of unaltered TILs, the stimulation of the patient’s own T-cells through peptide-based vaccines (35–37), and the use of TCR-mimic antibodies (13, 14). Therefore, the capacity to determine or predict the potential risk for off-target toxicities during the design and development of T-cell-based therapies is a major bottleneck for the broader use of these powerful immunotherapy approaches.

Unfortunately, there are no standard experimental methods that can be routinely applied to determine the risk of T-cell cross-reactivity in immunotherapies. Alanine scans, or X-scans, of the cognate peptide-target are usually performed to provide an initial assessment of T-cell cross-reactivity potential (38, 39). Such experiments do not directly provide information on potential off-targets, but can be used to guide proteomic searches for similar peptide motifs (40). The more recent development of yeast/phage-display and other high-throughput methods is starting to enable the screening of larger libraries of putative peptide-targets, but broader use of these methods is still limited by the cost and time required for library construction (41). In addition, these T-cell-based screenings are not as useful in the case of peptide-based vaccines, since the T-cell clones responding to the immunization will be different for each immunized individual (e.g., private specificity) (21, 42).

On the computational side, early work has been done in the context of heterologous immunity between viruses, mostly focused on the identification of peptide sequence similarities underlying cross-reactivity events (23, 43–45). This led to the development of a few sequence-based methods for cross-reactivity prediction, which can be further divided into methods based on i) peptide sequence identity (e.g., alignment based methods such as Expitope and iCrossR) and ii) peptide biochemical similarity (e.g., “alignment-free” methods such as dGraph and sCRAP). Expitope and iCrossR rely on the combined use of i) a Levenshtein distance to recover proteome-derived cross-reactive candidates with up to 4 amino acid mismatches to the query (i.e., high sequence identity), and ii) the subsequent ranking/filtering of these candidates based on a “epitope presentation score” (e.g., combined score from prediction algorithms for multiple steps of the class I HLA pathway, including proteasomal cleavage, TAP transport, and HLA binding). The output is also annotated with mRNA expression data to indicate tissue distribution and abundance (46).

An alternative approach for ranking peptides based on biochemical similarity was implemented in dGraph (47), which uses physicochemical properties to connect similar peptides into a network graph. More recently, a hybrid approach named sCRAP (48) was proposed. It uses a biochemical similarity matrix for computing a similarity score against the entire human proteome, and filters the output based on maximum tissue expression and HLA-binding affinity (e.g., predictions from NetMHC4 (49), NetMHCpan4.1 (50), and HLAthena (51)). sCRAP also enables customizing the score to increase the contribution of specific peptide positions, which could be used to bias the search based on potential hotspots for TCR recognition. In fact, attention to TCR hotspots on the pHLA surface is supported by both computational and experimental research showing that T-cell cross-reactivity can involve peptides with very diverse sequences, as long as they share a few key structural/biochemical similarities that are driving T-cell recognition (21, 52–55). These observations have supported the development of structure-based methods for T-cell cross-reactivity prediction (56–60), which have been discussed elsewhere (21, 25).

Although each approach provides interesting contributions to T-cell cross-reactivity analysis, all these methods have notable limitations. Expitope, sCRAP and iCrossR rely on sequence-based HLA-binding prediction algorithms to define the “universe” of possible off-targets out of the entire proteome. In doing so, they add a layer of prediction and introduce potential biases to the cross-reactivity screening. For instance, it is well-known that the accuracy of HLA-binding algorithms varies widely across HLA alleles, being less reliable for less prevalent HLAs (61, 62). Furthermore, cases of T-cell cross-reactivity involving peptides with low sequence identity, which have also been reported (21, 52, 63), cannot be predicted by Expitope and iCrossR due to the limited number of allowed mismatches.

Tools such as dGraph do not limit the number of mismatches and produce a relationship network for a set of peptides based on overall biochemical similarity. However, dGraph was developed for antibody cross-reactivity prediction (e.g., with linear epitopes involved in allergies), and was not directly evaluated for T-cell cross-reactivity (47). In addition, it does not include a clear statistical threshold to determine the level of significant similarity, hence hampering the interpretability of predictions. The more recent sCRAP is also not limited to a given number of mismatches, and introduces the use of position-specific weights to try and account for the TCR-specific hotspots. However, these weights were pre-defined for the MAGEA3-specific T-cell, and there was no rationale for customizing those weights for other targets of interest (48). Finally, structure-based methods are still limited by the number of available structures and the computational cost of large-scale modeling, being generally unsuitable for proteome-wide screenings (21, 25).

In this context, we developed CrossDome, a tool that performs peptide screening on multi-omics data from healthy tissues and predicts the risk for off-target toxicity with unrelated self-derived peptides. By relying on experimentally determined data from real pHLA complexes (i.e., immunopeptidomics data from mass-spectrometry studies), we overcome the potential biases of sequence-based HLA-binding prediction algorithms. Our approach leverages a large dataset of amino acid’s biochemical properties, allowing us to predict peptides that are biochemically similar to desirable targets, without enforcing any sequence identity cut-off. In addition, we demonstrate how structural data on TCRpHLA interactions can be used to tailor CrossDome predictions in order to account for TCR-specific hotspots. Finally, we compute a p-value for each putative cross-reactive target, therefore providing statistical support to estimate the off-target toxicity risk associated with each prediction. We further improve the significance of our results by integrating functional data to evaluate expression level, HLA-binding, and immunogenicity of putative off-targets, which can help prioritize candidates for experimental validation. CrossDome is released as an R package with support for technical and non-technical users, enabling both lower level control and a user-friendly application for users without coding experience.

## 2 Material and methods

### 2.1 Collecting reference experimental datasets

Immunopeptidomics data on naturally occurring self-derived peptides was retrieved from several sources, including HLA Ligand Atlas (64), HLAthena (51), SysteMHC (65), IEDB (66), and two other published datasets (67, 68). The data was filtered considering only 9-mer peptides presented by Class I HLA alleles (**Supplementary Figure S1A**). Next, we combined data sources into a non-redundant local database, organized by HLA allele restriction. In turn, each allele produces a single background database for the CrossDome screening algorithm. In order to evaluate the false-positive ratio, we also collected published data on 16 peptides previously identified as T-cell cross-reactivity off-targets for one of four well-known tumor-associated antigens: MAGEA3 (27, 41, 69, 70), NY-ESO-1 (69), TMEM161A (71) and AFP (72) (**Supplementary Table 1**). Finally, we also obtained a dataset of 60 synthetic peptides experimentally-determined to be recognized by the A3A TCR, being therefore cross-reactive with the cognate peptide from MAGEA3 (**Supplementary Figure S1B**). This data was produced by a yeast-display screening experiment previously reported by Gee et al. (2018) (73), and includes peptide sequences with up to 6 mismatches in relation to the cognate peptide (i.e., only 33% sequence identity). We also complemented this dataset with known off-targets of MAGEA3 (e.g., TITIN and MAGEA6). The full set of MAGEA3 off-targets is available in the CrossDome repository on Github.

### 2.2 Implementing a new similarity model based on biochemical profiles

The biochemical properties of amino acids have been used in previous work to estimate the similarity between peptides or protein binding motifs (44, 45, 74, 75). Here, we used a library with over 500 biochemical properties from AAIndex (76), a gold standard database of amino acids properties. This database has been used as a source of amino acid features for machine learning (77), and was used here to implement a new model to measure peptide similarity. First, the AAIndex data were summarized by using principal component analysis (PCA). The biochemical properties were then summarized into 12 principal components, holding 95% variance in the dataset (**Supplementary Figure S2**). The resulting eigenvectors were converted into a matrix of biochemical properties, spanning the 20 natural amino acids.

Each peptide was represented using this matrix, hereafter referred to as a biochemical profile (BP). In turn, the biochemical profiles of two different peptides can be used to compute a distance between these peptides, named as relatedness score (RdS). In order to compute this relatedness score, we implemented a weighted Euclidean distance. The weighted vector can be derived from TCR hotspots in the peptide sequence, i.e., position-specific weights related to known bonds/interactions between TCR and peptide molecules. This implementation penalizes biochemical profiles that deviate in hotspot positions. The relatedness score was normalized by peptide length, where low values represent highly similar peptide pairs (i.e., stronger candidates for cross-reactivity). Note that in its current implementation, our algorithm is limited to the analysis of 9-mers, which account for most of the peptides displayed by Class I HLA alleles (51). Finally, we compared the performance of the biochemical-profile-based approach with evolution-based substitution matrices. This revealed the extent to which our BP-based approach can capture peptide similarities beyond what would be found with a standard substitution matrix (e.g., BLOSUM (78)), keeping all other parameters equal (e.g., same query, Page 3positive control, and universe of peptides). The comparison was performed using Biostrings, an R Bioconductor package (79).

### 2.3 Monte Carlo simulation of peptide pairs and statistical validation

Statistical thresholds are essential to determine confidence levels in computational analysis, but no reference thresholds have been provided in previous methods for T-cell cross-reactivity prediction. To determine confidence levels in CrossDome predictions, we conducted a Monte Carlo simulation using peptide pairs derived from our immunopeptidomics database. The analysis was designed to produce 5 million simulated pairs, covering a wide range of class I HLA alleles. Next, an individual relatedness score was calculated for each peptide pair, using the aforementioned methods. The resulting RdS distribution was tested using the Shapiro-Wilk test (80). Then, we utilized the “*p*-norm” function to derive probability values for each peptide pair. Finally, the statistical threshold was defined based on the highest p-value associated with any of the experimentally-validated peptide pairs (**Supplementary Table 1**). This procedure allowed us to determine the relatedness score sensitivity to identify “real” cases within the background noise.

### 2.4 Uncovering TCR-peptide interactions from structural data

X-ray crystallography data for the A3A/MAGEA3/HLA-A*01:01 TCRpHLA complex was retrieved from the Protein Data Bank (81) to determine the molecular interactions between the engineered TCR and the cognate MAGEA3-derived peptide (PDB ID: 5BRZ) (70). The crystal structure was processed and cleaned using the PDBFixer tool from OpenMM suite (82). Hydrogen atoms were included assuming neutral pH (pH = 7.0), using the CHARMM36 force field parameterization protocol (83). The GetContacts package (84) was used to derive TCR-peptide interactions from the 3D structure, which we used to create TCR contact maps (CM). All interactions supported in this package were included. The hydrogen bond threshold was changed to 4.0 A, following the parameters used for curated TCR contacts on IEDB (85).

Two different CMs were produced for the same TCR in this analysis: CM-crystal and CM-custom. On the one hand, CM-crystal was obtained by performing a per-peptide-position cumulative sum of contacts derived from the reference crystal structure with GetContacts. These per-peptide-position contacts were then converted into a frequency vector, and pseudo counts (penalty = 0.5) were included in positions without TCR interactions. The resulting penalty vector for CM-crystal had the following values: w = (3.0, 0.5, 0.5, 4.0, 2.0, 0.5, 1.0, 1.0, 0.5). CM-custom was designed with knowledge-based weights informed by both data derived from the molecular dynamics simulation, and data regarding HLA binding requirements. We performed a 100 ns molecular dynamics simulation using the Gromacs 2021.2 package (86). The resulting data was divided into distinct time points for analysis, starting with the input structure (i.e., static data from crystal) and extending in 10 ns increments from the simulation (i.e., dynamic data). A stride step equal to 50 was adopted to recover frames from the simulation, which were used as input for GetContacts in order to obtain both the type and frequency of TCR-peptide interactions. Since the molecular dynamics simulation also enables energy calculations that are not available in GetContacts, we manually accounted for the occurrence of short-range Coulomb interactions with TCR residues (**Supplementary Figure S3**). Finally, we also accounted for the importance of peptide positions 3 and 9 for binding to HLA-A*01 alleles, as described in the SYFPHEITHI database (87). The resulting empirical penalty vector for CM-custom had the following values: w = (3.0, 0.5, 2.0, 4.0, 2.0, 0.5, 1.0, 1.0, 2.0).

### 2.5 Integrating functional data and third-party predictions

The clinical relevance of each candidate predicted by CrossDome might depend on additional functional properties, such as off-target expression and immunogenicity (i.e., capacity to trigger T-cell response) 8 (46, 48, 69). Therefore, to provide additional support for target prioritization, we incorporated into CrossDome results data from i) gene expression, ii) HLA binding affinity, iii) and peptide immunogenicity.

Transcriptomics data was retrieved from Human Protein Atlas (88), and highlights two essential aspects for the source antigen: i) abundance, which indirectly affects the number of HLA-displayed peptides at the cell surface, and ii) localization, which allows characterizing a candidate profile as tissue-specific or ubiquitous. We collected 37 healthy tissues spanning mRNA expression for 25,000 coding genes. To mitigate discrepancies from transcript to protein expression, the expression levels were normalized to transcripts per million protein-coding genes (pTPM) for healthy tissue in the database. Next, we classified the gene expression using the TissueEnrich package (89). The genes were separated into five groups: tissue-enriched, tissue-enhanced, group-enriched, low-tissue-specificity, and not-expressed profiles.

Finally, all eluted peptides included in our reference database were annotated with predicted immunogenicity (e.g., immunogenicity score) and predicted HLA binding (e.g., predicted IC50 binding affinity), for each included HLA Class I allele. HLA binding affinities were predicted with MHFlury 2.0 (90), which also accounts for intracellular peptide processing. Immunogenicity values were predicted with a recently published method called DeepImmuno (77). Taken together, these data can provide additional “clues” regarding T-cell toxicity and tolerability levels (69, 91). It is worth highlighting that our approach to off-target prediction differs from previous methods in that we do not rely on machine learning algorithms to identify “real” HLA-binders. Instead, we use these methods as additional criteria for prioritizing high-risk candidates.

### 2.6 Software development and web application

An overview of the CrossDome algorithm is provided in **Supplementary Figure S4**. In summary, for a peptide-centered prediction CrossDome requires as input only a peptide sequence, and its HLA allele restriction. Currently, CrossDome does not search across different HLA alleles, so the universe of self-derived peptides is restricted to the HLA allele of interest. For a TCR-centered prediction, the user would also provide the TCR contact map.

After ranking the peptides based on the relatedness score, CrossDome annotates each predicted off-target with a calculated p-value, and generates multiple plots with additional information on gene-expression, HLA binding and peptide immunogenicity. For software development, we adopted the R language (version 4.4) and S4 object-oriented pattern. To ensure best practices, we leverage R development guidelines, including packages for software design, testing, and documentation, such as devtools (92), usethis (93), testthat (94), and roxygen2 (95), respectively. Next, we create a user-friendly application using the Shiny framework (96). The web application was built upon an interactive interface to produce data visualization and searchable data tables.

## 3 Results

### 3.1 Immunopeptidomics data can be leveraged for off-target toxicity prediction

**Figure 1:**
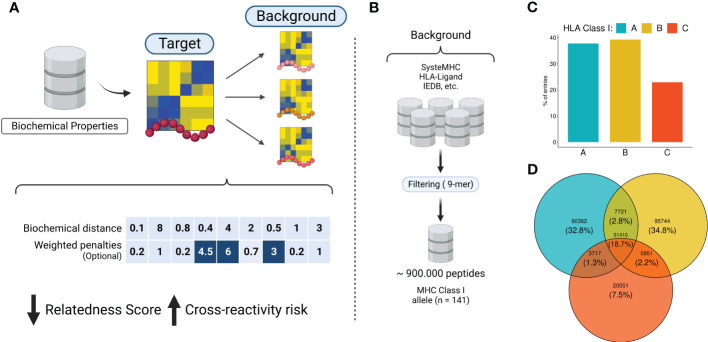
The rationale behind the biochemical approach implementation. **(A)** Biochemical properties from AAIndex are summarized using dimensionality reduction techniques. The eigenvectors are utilized to convert peptide sequences into biochemical profiles. Next, similar peptides are screened in the immunopeptidomics database. Each pairwise comparison produces a distance-based metric called relatedness score (RdS), which is used for peptide-centered predictions. The relatedness calculation can optionally utilize a position-specific weighted vector, for TCR-centered predictions. Higher values are related with strong penalties (e.g., positions in dark blue). **(B)** A comprehensive immunopeptidomics database was created by leveraging several public datasets. **(C, D)** Quantitative description of HLA alleles in the CrossDome database. Image created with BioRender.com.

Currently, the assessment of T-cell off-target toxicity risk in clinical and research settings is limited and heavily dependent on the accuracy of sequence-based HLA binding prediction tools. This dependence on additional layers of prediction can significantly increase the risk of false positives (97, 98). CrossDome, on the other hand, addresses this issue by screening for potentially cross-reactive peptides within “real” peptides, experimentally-determined by immunopeptidomics assays. This approach reduces the number of spurious candidates, and minimizes potential biases of HLA binding prediction tools. First, the methodology described in section 4.2 allowed us to convert peptide sequences into biochemical profiles (**Figure 1A**), which were in turn used to calculate distances between peptides (e.g., RdS). Using the RdS, we can perform pairwise comparisons between a tumor-associated query peptide, and a large reference dataset of self-derived peptides from immunopeptidomics databases. We created a reference dataset by retrieving over 900,000 eluted peptides from five different databases, covering 141 HLA Class I alleles (**Figure 1B** and **Supplementary Figure S1A**). In this combined reference database, HLA-B-restricted peptides are the most prevalent (40%), followed by HLA-A (38%) and HLA-C (22%) (**Figure 1C**). Although some peptides are shared among loci (**Figure 1D**), the large majority of the displayed peptides are HLA-exclusive.

### 3.2 CrossDome’s relatedness score outperforms alignment-based methods

**Figure 2:**
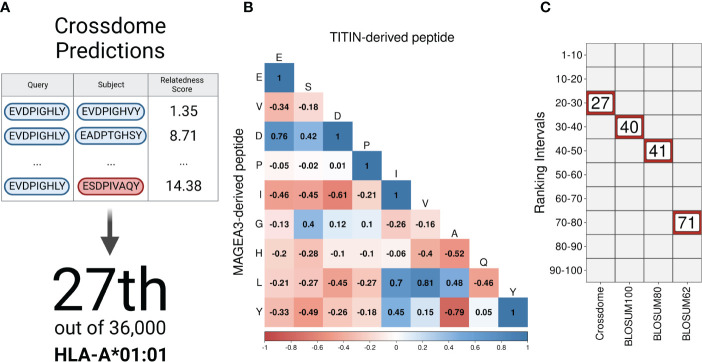
CrossDome performance against traditional approaches. **(A)** Evaluating CrossDome using MAGEA3-TITIN. Titin-derived peptide was recovered at position 27th out of 36,000 eluted peptides. **(B)** Residue-level comparison using Pearson correlation test. **(C)** Ranking-related heatmap displaying MAGEA3-TITIN positioning across distinct methods.

As a proof of principle for CrossDome, we used the tumor-associated peptide from MAGEA3 (EVDPIGHLY) as a query, and evaluated the capacity of our method to recover the known cross-reactive peptide derived from TITIN (ESDPIVAQY) among the top-ranked putative off-targets. Using the aforementioned relatedness score, the TITIN off-target was predicted at the 99+ percentile rank, at position 27 out of 36,000 peptides displayed by HLA-A*01:01 (**Figure 2A**). The list of best-scored peptides reported by CrossDomealso included MAGEA3 paralogs, other validated off-targets, and a few other highly similar peptides (**Supplementary Table S3**). In addition to the relatedness score, we measured residue-level correlation among the two peptides. It allowed us to evaluate the peptide composition and correlation between biochemically similar residues. In general, the MAGEA3-TITIN pair shows a strong correlation (Pearson p-value ≤2.2*e* ^-16^). **Figure 2B** diagonal displays the residue-based correlation among MAGEA3 and TITIN-derived peptides. Note that highly correlated residues recapitulate expected amino acid biochemical similarities. For instance, we observe high correlation between polar residues with the same charge (e.g., glutamic acid and aspartic acid), and between non-polar residues (e.g., leucine and valine), while we see low correlation between polar residues with opposite charge (e.g., aspartic acid and histidine).

However, our implementation of the relatedness score goes beyond these obvious associations when determining peptide similarity. To demonstrate this point, we compared our ranking based on the relatedness score with alternative rankings based on traditional alignment-based metrics (e.g., substitution matrices). Keeping all other factors equal (e.g., same query and same reference universe of peptides), the highest BLOSUM matrix (i.e., BLOSUM100) placed the TITIN off-target at the 40th position. Higher numbers on BLOSUM matrices are more accurate for highly similar sequences, therefore not reasonable to predict cross-reactivity between unrelated peptides (99). On the other hand, BLOSUM62 should provide higher sensitivity for low-similarity peptides, but it displayed an even worse performance in our experiment, ranking TITIN at the 71st position. **Figure 2C** shows a ranking comparison between searches using the relatedness score, or substitution matrices. In fact, the relatedness score implemented in CrossDome outperformed alignment-based metrics regarding both sensitivity, as observed by the ranking of the TITIN-derived peptide, and computational performance, as the average run-time is 15 times faster than sequence alignment. Moreover, unlike sequence alignment methods, our approach does not depend on parameters such as gap and mismatch penalties.

### 3.3 A statistical threshold can be used to estimate off-target toxicity risk

**Figure 3:**
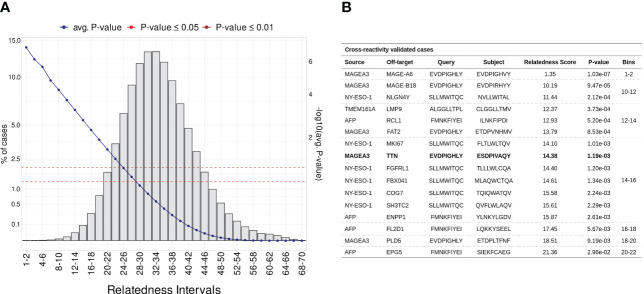
Establishing a statistical threshold for relatedness distribution. **(A)** Benchmarking putative cross-reactivity pairs in allele agnostic simulation. In total, 5 million peptide pairs were simulated and categorized based on relatedness intervals. For each pairwise comparison was calculated an empirical p-value. The left-sided y-axis shows the percentage of cases per bin (i.e., intervals). The blue line represents the average p-value in each interval (second y-axis). Additionally, red and brown are associated with standard p-value thresholds, 0.05 and 0.01, respectively. **(B)** Table covering experimentally validated CR cases. MAGEA3-TITIN was retrieved at 14-16 bin, which holds less than 0.1% of putative cases.

Considering that most documented cases of T-cell off-target toxicity have been associated with molecular mimicry (20, 21, 52, 70), we expected that validated target/off-target pairs should in general present low relatedness scores. In this context, the relatedness distribution obtained in the Monte Carlo simulation described in section 4.3 can both be used to understand the dispersion of validated cases, and to identify confidence boundaries in our predictions (i.e., statistical threshold). As a result, we obtained a RdS distribution that largely resembles a Gaussian (u = 32.77, sd = 6.05, Shapiro-Wilk test ≤ 0.05). Next, we divided the distribution into intervals (i.e., “bins”) encompassing worse to best-scored peptide pairs (**Figure 3A**). As expected, the bins with low relatedness score values (i.e., best-scored cases, Bin ≤ 16), were highly populated with experimentally validated cases (**Figure 3B**;. **Supplemantary Table S1**).

For instance, the peptide pair related to the MAGEA3-MAGEA6 cross-reactivity was ranked among the most meaningful values (RdS = 1.35, p-value ≤1.03*e* ^-02^). MAGEA6 belongs to the melanoma-associated antigens, a paralog group with high sequence similarity (100). The MAGEA6-specific peptide deviates from MAGEA3 by a single conserved residue substitution at position eight (leucine to valine). Bin 14-16 holds the largest number of validated cases (n = 7), including the MAGEA3-TITIN pair. Finally, the AFP-EPG5 pair represents the last detectable cross-reactivity event (RdS = 21.36, p-value ≤2.96*e* ^-02^). The standard threshold for CrossDomepredictions was defined as p-value ≤ 0.01, based on this RdS distribution.

### 3.4 Contact maps can be inferred from structure and used to refine predictions

**Figure 4:**
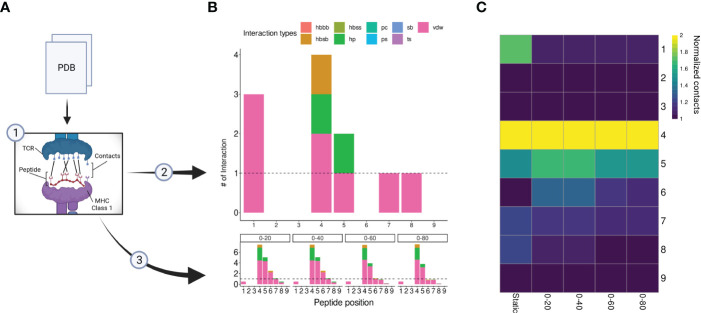
A3A-TCR contact maps construction. **(A)** A flowchart representing the contacts/interactions analysis. TCRpMHC interactions are depicted as black lines. The A3A crystal was submitted to two analyses (2), static, and (3) dynamic (molecular dynamics). **(B)** Each analysis produces a contact map summarized by peptide positions. The interaction types and frequency were retrieved using getcontacts. In total, nine interaction types can be detected, including hydrogen bond-related (hbbb, hbsb, hbss); salt bridge (sb); pi-cation (pc); pi-stacking (ps); t-stacking (ts); hydrophobic (hp); van der Waals (vdw). The dynamic contact map was summarized into four distinct time points: 0-20, 0-40, 0-60, and 0-80 nanoseconds. The overall profile showed similar hotspots compared to the static map. **(C)** Normalized contacts are displayed on the heatmap. Image created with BioRender.com.

Although peptide similarity can provide us with a baseline probability for observing T-cell cross-reactivity, different cross-reactivity patterns can be observed for different T-cell clones (21). In other words, cross-reactivity is ultimately determined by the particular T-cell clone that is tested, and by the TCR-specific interactions with the cognate pHLA complex (52, 53). Here, we investigated the possibility of refining the CrossDomesearch in order to provide a TCR-specific off-target toxicity prediction. For that, we derived the molecular interactions between an engineered TCR (A3A) and its cognate MAGEA3-derived peptide-target from the available crystal structure of the TCRpMHC complex (**Figure 4A**). The contact map revealed a high preference for peptide positions 1, 4, 5, 7, and 8 (**Figure 4B**). Positions 4 and 5 showed higher interaction type diversity, including van der Waals, hydrogen bonds, and hydrophobic interactions.

Our A3A-derived contact map was consistent with curated data from IEDB-3D (85), and produced promising results when used to tailor CrossDome predictions for MAGEA3. Therefore, it supported our idea that the weights for CrossDome predictions can be derived from a reference TCRpHLA structure (i.e., with the cognate pHLA). However, we reasoned that significant differences could appear between contact maps derived from static (e.g., a single crystal structure) and dynamic sources (e.g., data derived from NMR experiments or molecular dynamics simulations). To investigate that, we calculated contacts for distinct time points in a 100 ns long molecular dynamics simulation (**Figure 4B**). In the overall profile obtained with the GetContacts package, position-specific preferences displayed by contact maps from molecular dynamics were highly similar to that of the crystal, including interaction types. However, a few interactions were lost, related to positions 1, 7, and 8 (**Figure 4C**). The reduction of TCR interactions over the simulated time might indicate these peptide positions were more involved with HLA interactions. In the case of position 1, we were still able to detect short-range Coulomb interactions with TCR residues in the simulation (**Supplementary Figure S3**). This type of interaction is not supported by GetContacts, but it can be computed with the gmx energy tool from Gromacs.

### 3.5 CrossDome predictions are consistent across protocols

**Figure 5:**
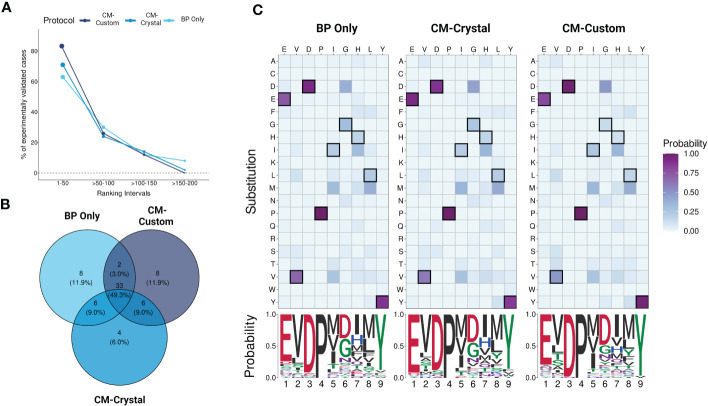
Evaluating protocols using yeast-displayed peptides. **(A)** Overall performance across CrossDome protocols. The y-axis corresponds to experimentally validated off-targets. The data was filtered considering best-scored candidates (p-value <0.01). The ranking was divided into four intervals. In total, three screenings were carried out, i) Biochemical properties (BP) only, ii) A3A-derived static contact map, and iii) manually curated contact map. **(B)** Venn diagram showing TOP50 best-scored peptides overlap among protocols. **(C)** TOP50 substitution heatmaps and seqLogos. The heatmaps present the substitution probability for each position across candidates. The seqlogos summarizes the most prevalent residue per position. The amino acid colors are related to biochemical classifications.

CrossDome can predict T-cell cross-reactivity using two distinct approaches: i) based only on the biochemical profile (BP) of the peptides, or ii) using a combination of peptide’s biochemical profile and TCR contact map (BP + CM). Note that in the first case we have a peptide-centered prediction, regardless of TCR information. While, on the second case, we have a TCR-centered prediction. To evaluate the overall performance of CrossDome using these different protocols, we leveraged a dataset of over 60 unrelated peptides known to be cross-reactive with MAGEA3, as described in section 2.1. As expected, the CM-based predictions increased the overall number of experimentally validated cases under statistical confidence (**Figure 5A**). Moreover, we could observe an incremental increase in sensitivity among protocols. The percentage of experimentally-validated cases in the top 50 ranking was equal to 63%, 71%, and 82% for BP only, CM-crystal, and CM-custom, respectively.

Subsequently, we analyzed qualitative changes in each protocol regarding the top 50 off-target candidates (**Figure 5B**), which were broadly shared between protocols (n = 33). The high level of agreement between protocols demonstrates CrossDome’s accuracy even without the CM imputation. Further, at the sequence level, we noticed a reduction in the substitution rate at peptide positions 1, 3, and 9. Specifically, top ranking peptides on the CM-based screenings showed an even greater conservation of glutamic acid, aspartic acid, and tyrosine at these positions (**Figure 5C**), reflecting differences in the weighted vector values in each analysis (see section 2.4). The glutamic acid (position 1) displayed the largest increase across protocols, ranging from 74% up to 92% in residue conservation. The ranking of the known TITIN-derived cross-reactive peptide also improved between protocols, from the 27th position on BP to 8th and 6th position on CM-crystal and CM-custom, respectively. Together, these findings validate the contact maps as a reliable resource to tailor CrossDome predictions for a specific TCR.

### 3.6 CrossDome outperforms sCRAP when predicting known off-target toxicity cases

We used the aforementioned dataset of 16 validated cross-reactive peptide pairs (**Figure 3B**) to compare the top-ranking predictions by CrossDome with those of the recently published sCRAP tool (48). The same 4 cancer-associated antigens were used as queries for each tool (**Supplementary Table S1**), with default protocols. On CrossDome, we used the BP-based prediction (i.e., no TCR-based position-specific weights). On sCRAP, we used recommended settings (i.e., including default position-specific weights). In this context, CrossDome was able to predict 10 out of 16 off-targets within the top 50 sequences. For the other 6 cases, while not in the top 50, CrossDome still predicted the off-target at the 99+ percentile rank (**Figure 3**). sCRAP, on the other hand, was only able to predict 5 out of 16 within the top 50, and we could not determine the percentile rank for the other predictions since only the top 100 entries are provided.

Note that a fair direct comparison between peptide rankings obtained with these tools might not be possible, since they differ in multiple aspects (e.g., different search algorithms, different reference universe of self-derived peptides, different use of third-party methods, etc.). Instead, we decided to evaluate if there was any overlap between the top-ranking predictions by CrossDome and sCRAP in these experiments. To determine this overlap, we used only the top 50 predicted peptides by each tool, for each query (**Supplementary Table S2**). Any agreement between tools with such different implementations highlights their potential to identify dangerous off-targets. However, it is important to note that the entire list of candidates predicted by CrossDome is comprised of real immunopeptidomics-derived peptide targets. In addition, with the exception of the MAGEA3 prediction, multiple of these off-targets are also predicted to be both immunogenic and strong HLA-binders. In spite of that, only a fraction of these off-targets is also predicted by sCRAP. This difference is probably driven by the fact that sCRAP predictions are highly populated by predicted peptides, for which there is no available immunopeptidomics validation. Interestingly, in the case o MAGEA3, only the TITIN-derived peptide was kept as a candidate target after considering HLA binding and immunogenicity predictions. On the other hand, 12 of the top 50 predictions by CrossDome were also in the top 50 by sCRAP, with no further support to exclude them. The full list of top 50 predictions for both tools can be found in **Supplementary Table S3**.

### 3.7 MAGEA3-specific predictions can be refined based on mRNA expression and immunogenicity

**Figure 6:**
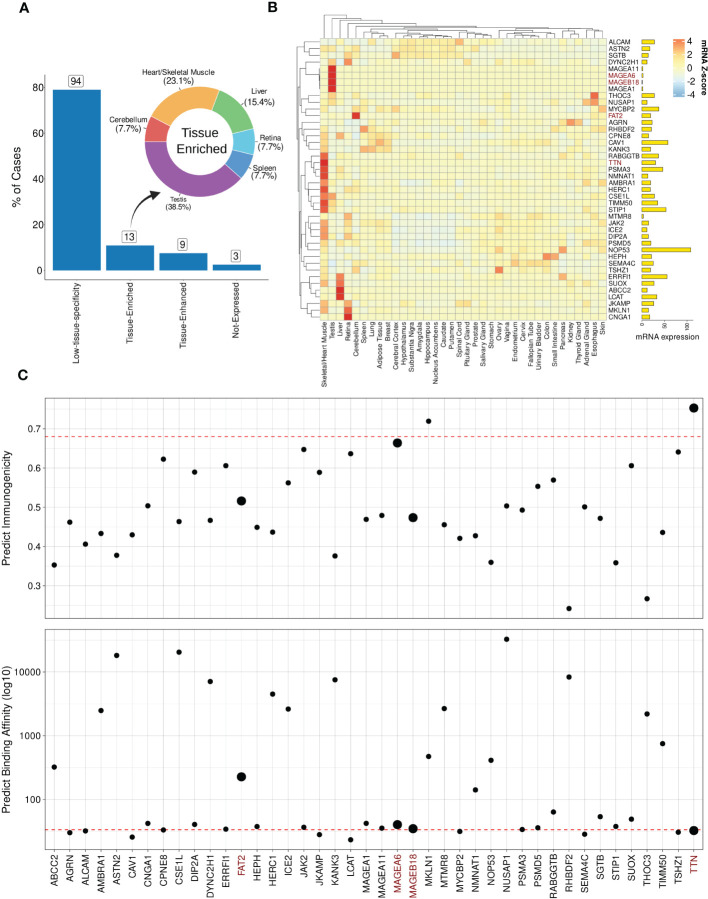
Expression and immunogenic profiles related to the cross-reactive candidates. **(A)** Tissue specificity groups across all best-scored candidates (p-value < 0.01). The donut plot displays tissues/organs related to “Tissue-enriched” candidates, i.e., genes with tissue-biases expression. **(B)** Heatmap with TOP50 best-scored peptides showing Z-score mRNA expression (pTPM). High (red) to low (blue) expression values on color key. Finally, total mRNA expression is represented on bar plot. **(C)** Peptide immunogenicity and MHC-antigen binding affinity predictions. The red dashed line represents the MAGEA3-derived peptide (EVDPIGHLY). The data points size reflects experimentally validated candidates. TTN corresponds to TITIN gene aliases.

In order to demonstrate the impact of incorporating mRNA expression for off-target localization and tolerability assessment, we applied this additional analysis to the best-scored peptides derived from the MAGEA3 screening using BP-based protocol. Note that a few peptides were dropped due to lack of similarity with RefSeq Protein database (101). **Figure 6A** shows the expression profile summary across all best-scored peptides (RdS P-value ≤ 0.01). The MAGEA3 screening presented higher percentages associated with low-specificity followed by tissue-enriched, tissue-enhanced, and not-expressed groups. In the tissue-enriched group, several candidates are strongly associated with the Heart/Skeletal Muscle (TITIN, TIMM50, PSMA3, etc.), Testis (MAGEA6, MAGEA11, etc.), Liver (LCAT, ABCC2, etc.), and Cerebellum (FAT2). Next, we displayed the expression levels for the top 50 peptides (**Figure 6B**). The experimental relevant MAGEA3 off-targets, TITIN and FAT2, were correctly assigned to respective tissues (41, 102, 103). Curiously, other liver-biased candidates identified here, such as LCAT, were not associated with off-target toxicity in previous clinical trials (26).

Next, we hypothesized that genes with ubiquitous (low-specificity) expression could be considered dangerous candidates due to a putative broad autoimmune response. For instance, NOP53, a ribosome biogenesis factor, showed a high expression level across all tissues (avg. PPM expression = 317.32). Furthermore, in terms of biochemical similarity, the NOP53-related peptide showed a relatedness score equal to 12.95. However, the NOP53 peptide (EVAPAGASY) has no evidence of T-cell positive assays on IEDB (66). The lack of T-cell assays reporting NOP53 can be indicative of either a low immunogenicity profile (e.g., low binding HLA affinity or lack of immunogenic features), or potential immune tolerance mechanisms.

To further investigate the immunogenicity potential of top-ranked putative off-targets, we conducted computational predictions of both HLA binding and peptide immunogenicity (**Figure 6C**). MAGEA3-specific predictions for HLA-binding (BA = 33.50) and immunogenicity (IS = 0.68) were used as references (dashed red line). In total, 23 off-target candidates were predicted as putative strong binders, i.e., IC50 ≤ 50 nM. Additionally, three candidates were reported with a meaningful immunogenicity level (e.g., > 0.65). Only two of the candidates were predicted to be both strong HLA-binders and highly immunogenic. As expected, TITIN has a similar binding affinity to the cognate MAGEA3 peptide (< 50 nM) with a superior immunogenic score (> 0.90), and would have been predicted as a dangerous off-target using our package. MAGEA6, another experimentally validated cross-reactive peptide, also displayed a similar profile. FAT2 and MAGEA18 had worse scores than MAGEA3 and other validated cases. The combination of high affinity (i.e., low predicted binding value) and high immunogenicity score can indicate the most dangerous candidates on CrossDome predictions.

### 3.8 Increased usability promoted through an R package and user-friendly interface

R is a well-established language in the bioinformatics community. To improve CrossDome’s usability, we developed an R package containing several functions for predicting, analyzing, and visualizing cross-reactivity risk. Currently, the package allows the screening of putative off-targets by selecting a peptide-target (query) and our immunopeptidome database (subject). This database can also be combined with, or replaced by, a customized database. The CrossDome immunopeptidome database includes peptide immunogenicity and binding affinity predictions across several HLA Class I alleles. On average, the CrossDome run takes less than 1 minute per allele in a workstation machine (e.g., Intel Core i7 Processor, 32GB RAM), therefore allowing for batch analysis using several peptide-targets across distinct HLA alleles.

**Figure 7:**
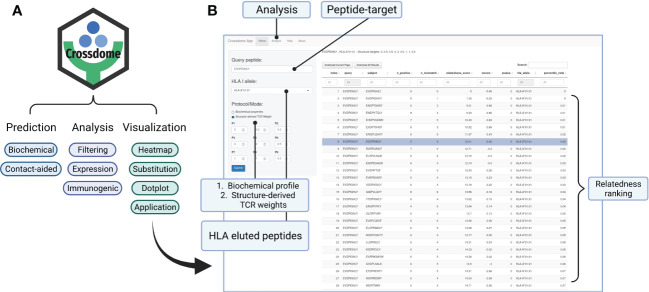
CrossDome R package. **(A)** Main features include expression heatmap, immunogenic plot, best-scored substitutions plot, and web application. **(B)** CrossDome user-interface built with R’s Shiny framework.

To foster reproducibility of results, we provided a tutorial/vignette series from basic usage to MAGEA3 analysis (**Supplementary Materials**). The MAGEA3 tutorial details contact map usage and calculation, including the comparison between BP and BP + CM predictions. In addition, the package was designed to store data reporting essential parameters and outputs in each execution. CrossDome results can be manipulated using dplyr, a well-known R package for data science (104), therefore promoting greater versatility for bioinformaticians and computational biologists. Finally, we developed a web application that allows CrossDome basic usage. Currently, the app generates an interactive table supporting filtering, ranking, and downloading (**Figure 7**).

## 4 Discussion

Here we describe CrossDome, a software suite for predicting off-target toxicity risk for T-cell-based cancer immunotherapies. Using experimentally-validated cases as positive controls, we demonstrate that for a given tumor-associated target of interest for immunotherapy, this implementation can identify self-derived peptides that represent a potential risk for off-target toxicity mediated by T-cell cross-reactivity. Therefore, our method reduces the screening space from many thousand peptides (e.g., entire host proteome) to dozens of high-risk candidates, also providing information about the immunogenic profile and tissue-specificity of these putative off-targets. More importantly, CrossDome goes beyond previously proposed methods by providing a p-value associated with each off-target prediction. Based on the computed distribution of known cross-reactivities involving cancer-associated antigens, the p-values can be used to define a significance cut-off for the off-target toxicity risk.

It is also important to note that most of the previously published methods for T-cell cross-reactivity prediction are based on a “target-centered” perspective. By considering properties of the peptide or the pHLA complex as the key to find potentially similar off-targets, these methods can provide a baseline prediction of cross-reactivity (46, 48, 57, 59, 105). However, different T-cell clones will express unique TCRs, which can have different specificities towards pHLA complexes (24, 53). In turn, this can be reflected in different cross-reactivity patterns/profiles among T-cell clones recognizing the same cognate peptide (21, 39, 63). In order to account for that, predictions with CrossDome can be performed using two alternative approaches: one based on the biochemical profile of the query peptide, and the other based on a combination of peptide’s biochemical profile and a TCR contact map. In the first case, we have the standard peptide-centered prediction, regardless of TCR information. This option is useful when the T-cell information is not available, or different T-cells can be triggered depending on the subject (e.g., peptide based vaccine design). Additionally, by changing the reference database, this approach can be used to identify relatedness between tumor associated antigens and microbial-derived peptides, therefore extending its applicability towards distance-to-self calculations on vaccine development studies (106–109). On the other hand, the second case provides a TCR-centered prediction (i.e., clone-specific off-target toxicity prediction). This option is preferred for users interested in the cross-reactivity profile of particular T-cell clones (e.g., in TCR-based immunotherapy). Tailoring CrossDome predictions with TCR information helps filtering out spurious candidates while recovering even more diverse peptide sequences, and mitigates the need for exhaustive search through experimental approaches (41, 52, 110). Consequently, CrossDome can reduce the time and costs associated with prioritizing antigens for T-cell-based immunotherapy, potentially accelerating their transition to clinical trials.

Our implementation choices on CrossDome are supported by extensive research on T-cell cross-reactivity, previously performed by us and by others. For instance, the role of pHLA structural similarity in T-cell recognition has been previously discussed, and even leveraged for cross-reactivity prediction (21, 58, 59, 111). It is also well known that T-cell recognition is driven by a few hotspots in the pHLA surface, and that T-cell cross-reactivity can be observed between peptides with very different sequences, as long as they share the same hotspots for TCR interaction (52, 63). Our work is also informed by previous implementations, which leveraged peptide sequence similarity, HLA binding prediction, and tissue expression patterns (46, 48, 105). Is worth noting that these tools rely on underlying AI-based methods for HLA-binding prediction, which have been a standard in the field. In addition, AI-based methods have enabled many other recent breakthroughs in biosciences (112). However, these methods were not yet successfully applied to the problem of T-cell cross-reactivity prediction, mostly due to the lack of large enough labeled training datasets. This landscape should change in the future, as data from high-throughput experimental methods for T-cell activation becomes more broadly available. In fact, available cognate TCR/peptide sequences from databases such as VDJdb (113) are already been leveraged to train AI-based models for the prediction of TCR specificity (69, 114–116). CrossDome can be used in combination with these methods to further accelerate the identification of peptide-targets and TCRs requiring experimental validation.

Different from previous methods, our tool does not rely on HLA-binding prediction to define the universe of self-derived peptides used in the search for off-targets. Instead, it relies on a local database of “real” peptides from immunopeptidomics studies. On one hand, this is a major advantage since it reduces false positive predictions (i.e., predicted off-targets that cannot be displayed by HLAs). On the other hand, this implementation restricts the universe to available experimental data, which might still be incomplete. Fortunately, immunopeptidomics has become a standard in the field, and we will continue updating our reference database as new datasets become available (65, 117). Note that CrossDome is also currently limited to the analysis of 9-mers, which account for most of the peptides displayed by class I HLA alleles. Future work will enable the expansion towards longer peptides, including those restricted to class II HLA alleles, therefore enabling cross-reactivity prediction for CD4+ T-cells. Although not associated with off-target toxicity, cross-reactivity involving these cells is a promising future direction due to newly discovered cytotoxic effects, and their role in mediating the production of autoreactive antibodies following vaccination (118, 119).

Another original component of our study relates to the demonstration of how structural information from a TCRpHLA complex can be used to derive a TCR contact map. Such contact map can be used by CrossDome as a per-peptide-position weighting system, enabling the aforementioned TCR-centered prediction of off-target toxicity. Note that automated extraction of the contact map from a TCRpHLA complex is not yet available on CrossDome, but it is a future implementation already being developed by our team. The best predictions in our TCR-centered experiments were obtained with a customized set of weights (CM-custom), considering dynamic contact maps, Coulomb interactions, and HLA binding motif. However, it is important to note that even the contact map derived from the TCR-peptide bonds detected on a single structure (CM-crystal) was already enough to recover over 71% of the validated cases in our dataset. Interested users can derive such static contact map from a growing number of crystal structures of TCRpHLA complexes being made available at PDB (81) and IEDB (66). Alternatively, we are also investigating if these contact maps can be accurately derived from 3D models, as new TCRpHLA modeling methods become available (120–123). If successful, this effort could enable future automation of structure-based contact map extraction from TCR sequences, such as those produced by single-cell TCR sequencing.

Finally, CrossDome can be easily incorporated into existing antigen discovery pipelines, therefore aiding the selection of better and safer peptide-targets and T-cell clonotypes for immunotherapy applications. The tool is under active development, and the beta version is available at https://github.com/AntunesLab/crossdome.

## Data availability statement

The original contributions presented in the study are included in the article/**Supplementary Material**. Further inquiries can be directed to the corresponding author.

## Author contributions

DA conceived the original idea behind this work. DA and AF designed the methods and experiments. AF selected the datasets, implemented the methods, and executed the experiments. DA and AF analyzed and interpreted the results. AF wrote the first draft of the manuscript. All authors contributed to the article and approved the submitted version.

## Supplementary material

Supplementary Figure 1 Background and validation datasets. (A) Schematic representation of sources used to build the reference self-derived background dataset for CrossDome. (B) Schematic representation of sources used for validation experiments.

Supplementary Figure 2 Dimensionality reduction of biochemical properties. (A) Scree plot presenting the explained variance per principal component (dimensions of the Principal Component Analysis). Twelve dimensions are accumulating 95% explained variance among AAIndex databases. (B) Two-dimensional plot displaying amino acid distribution according to the first and second principal components (dimensions) from the dimensionality reduction. Similar residues are grouped at closest distances, e.g., Isoleucine, Valine, and Leucine. Proline and Arginine are outliers.

Supplementary Figure 3 Histograms of Coulomb interactions. Short-range Coulomb interactions were calculated using the software gmx energy from Gromacs, and divided into intervals (x-axis). The energy reflects the interaction between the A3A MAGEA3-specific TCR molecule and each amino acid from the MAGEA3-derived peptide. The y-axis shows the fraction frame reporting that energy range (molecular dynamics). Low values are related to proximity, i.e., putative strong interaction with the TCR molecule. Positive values are related to repulsion from TCR molecular, potentially favoring MHC interaction.

Supplementary Figure 4 CrossDome workflow diagram. The required and optional inputs are displayed on top. Peptide and HLA allele parameters only support 9-mer sequences and Class I alleles, respectively. TCR contacts and known off-targets (e.g., positive controls) can be introduced as optional inputs. The “CrossDome screening” process is performed over a reference “universe” database, using the relatedness score to find biochemically similar peptides. Next, a statistical threshold (p-value < 0.01) and data integration are applied to the potential off-target candidates. The “Gene-donor expression” step maps mRNA expression data from genes to each candidate. The expression profile provides measurements across several healthy tissues. The “Tissue specificity plot” summarizes the expression profile and tissue specificity. Additionally, the “Substitution plot” provides insights into sequence substitution among the best-scored candidates. Finally, the “immune-related predictions” combine immunogenicity and binding affinity predictions for each candidate. The package functions are named below each step in the workflow.

Supplementary Table 1 Additional information on the 16 validated off target toxicity cases, involving 4 well-known tumor-associated antigens.

Supplementary Table 2 Overlap between sCRAP and the top 50 ranking by CrossDome, for 4 different queries.

Supplementary Table 3 Full list of peptides among the top 50 ranking for both sCRAP and CrossDome, for 4 different queries.
