# Supplementary figures and images for: CrossDome: an interactive R package to predict cross-reactivity risk using immunopeptidomics databases
Source: Front Immunol. 2023 Jun 12;14:1142573. doi: 10.3389/fimmu.2023.1142573 (PMC10291144; doi:10.3389/fimmu.2023.1142573)

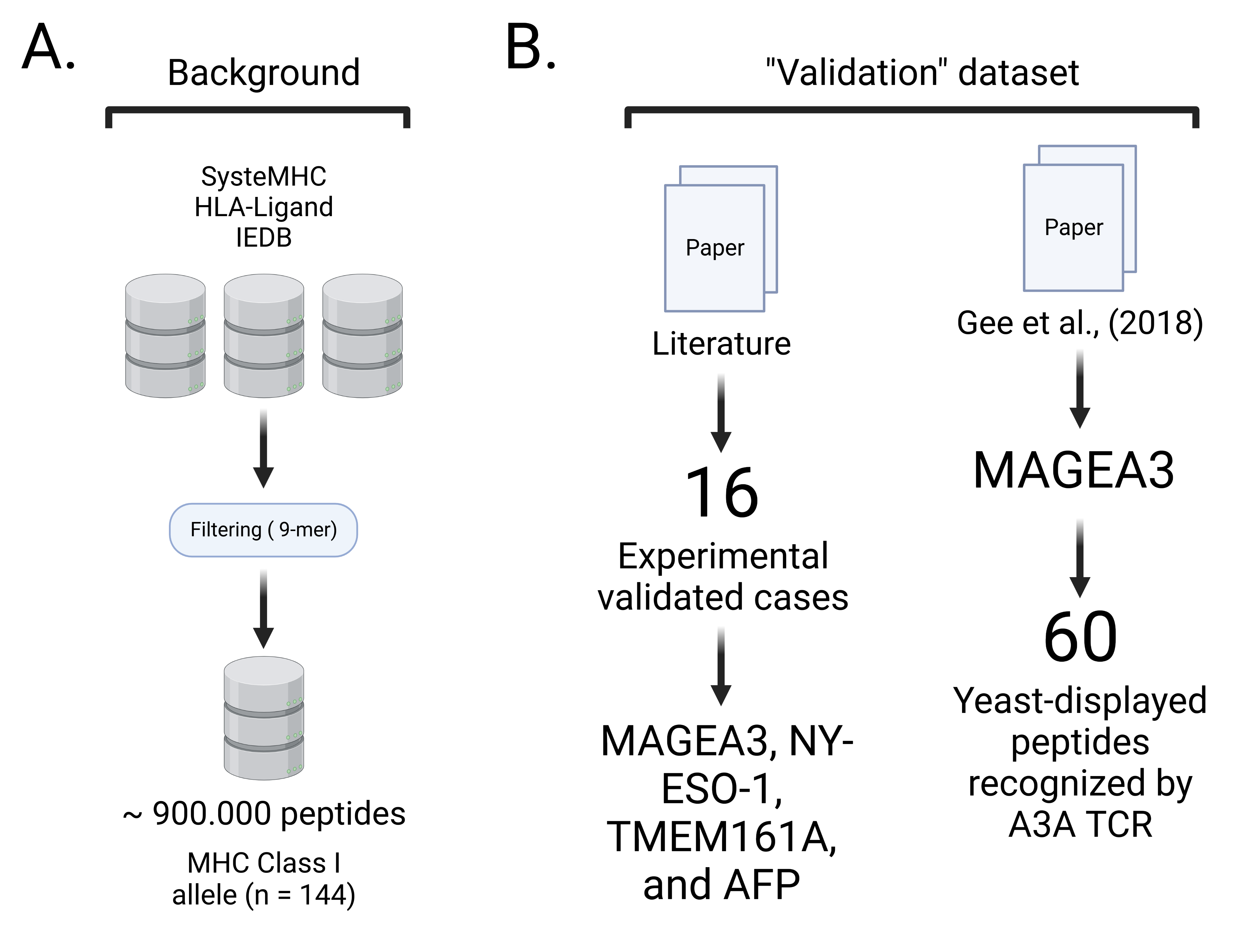

Supplement: Supplementary Figure 1 — Background and validation datasets. (A) Schematic representation of sources used to build the reference self-derived background dataset for CrossDome. (B) Schematic representation of sources used for validation experiments. [file Image_1.png]

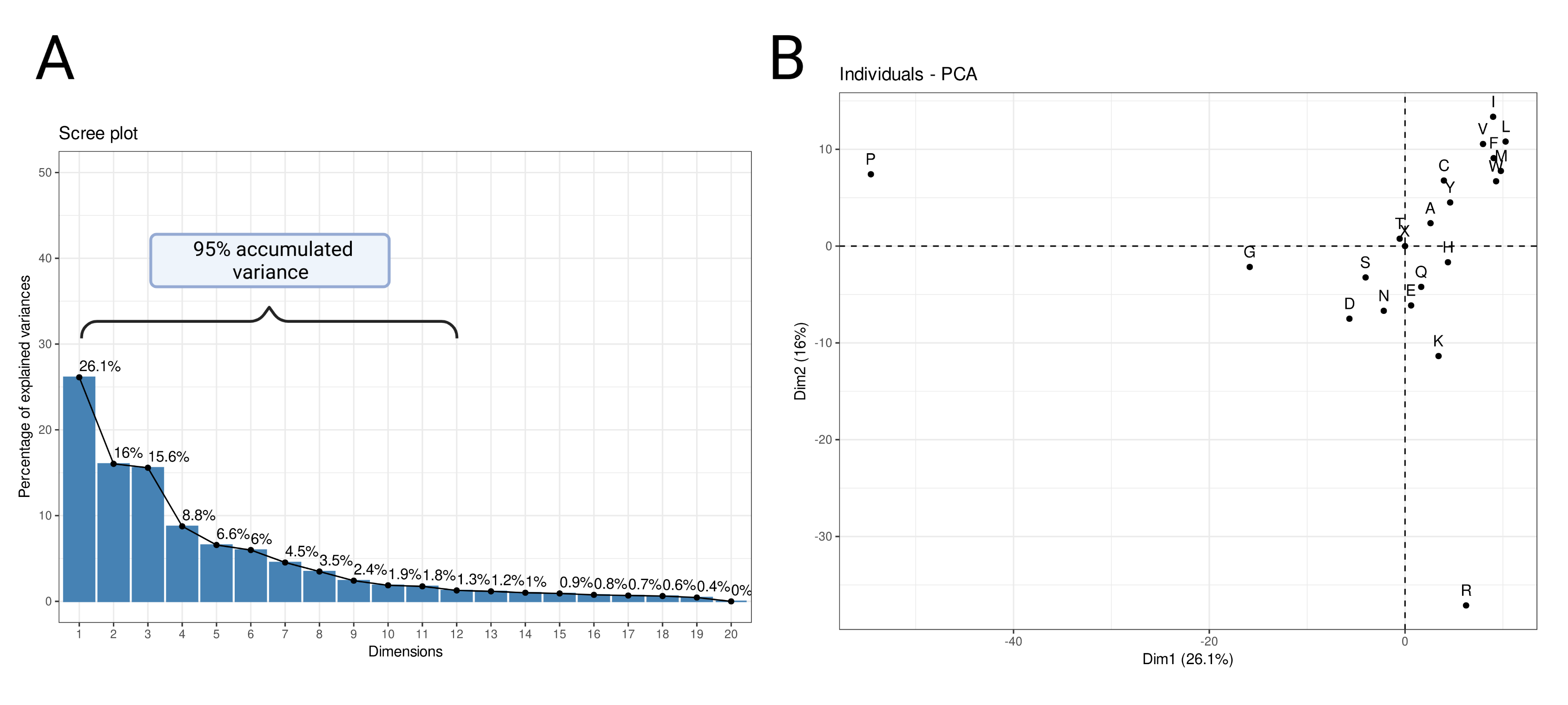

Supplement: Supplementary Figure 2 — Dimensionality reduction of biochemical properties. (A) Scree plot presenting the explained variance per principal component (dimensions of the Principal Component Analysis). Twelve dimensions are accumulating 95% explained variance among AAIndex databases. (B) Two-dimensional plot displaying amino acid distribution according to the first and second principal components (dimensions) from the dimensionality reduction. Similar residues are grouped at closest distances, e.g., Isoleucine, Valine, and Leucine. Proline and Arginine are outliers. [file Image_2.png]

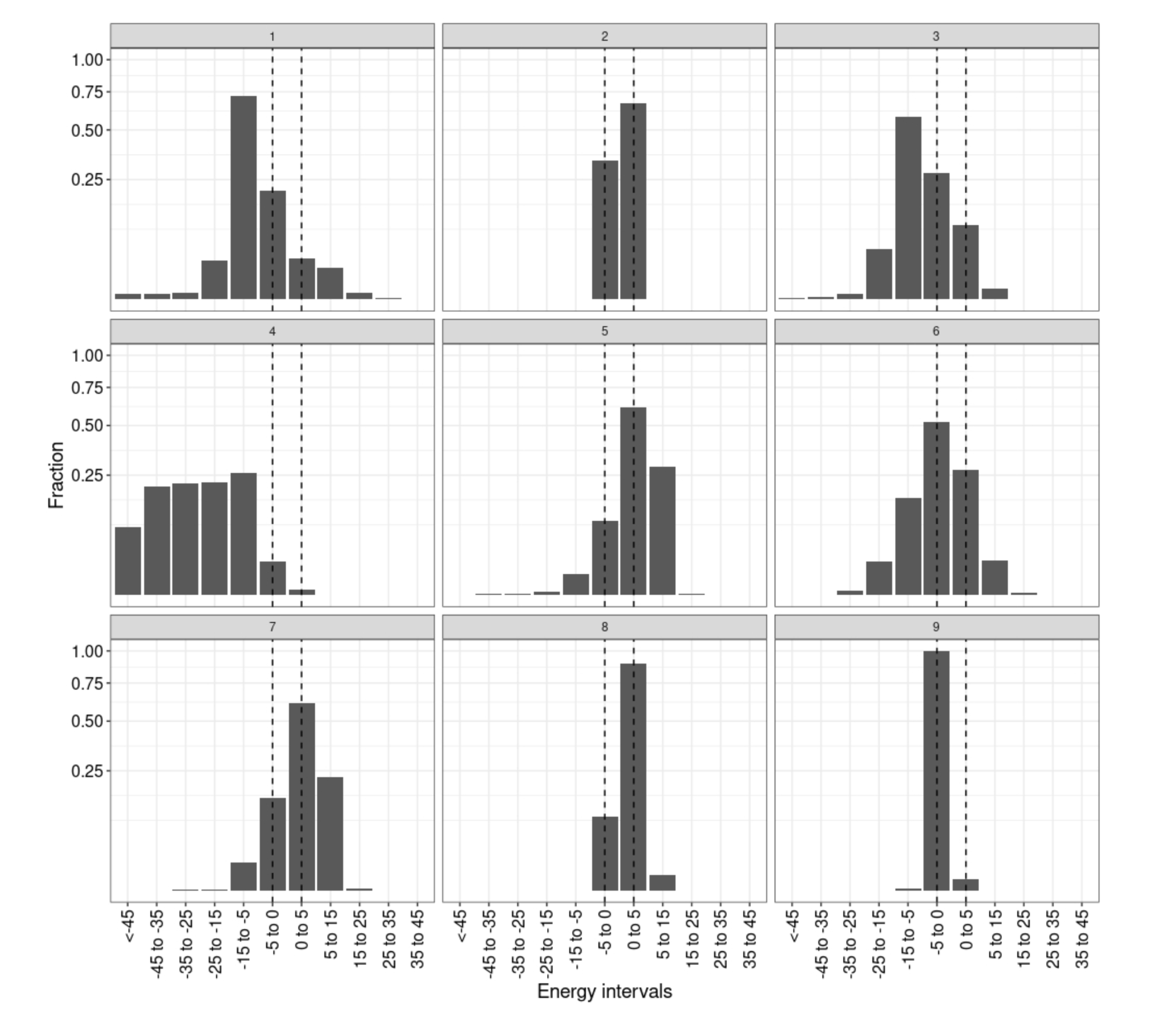

Supplement: Supplementary Figure 3 — Histograms of Coulomb interactions. Short-range Coulomb interactions were calculated using the software gmx energy from Gromacs, and divided into intervals (x-axis). The energy reflects the interaction between the A3A MAGEA3-specific TCR molecule and each amino acid from the MAGEA3-derived peptide. The y-axis shows the fraction frame reporting that energy range (molecular dynamics). Low values are related to proximity, i.e., putative strong interaction with the TCR molecule. Positive values are related to repulsion from TCR molecular, potentially favoring MHC interaction. [file Image_3.png]

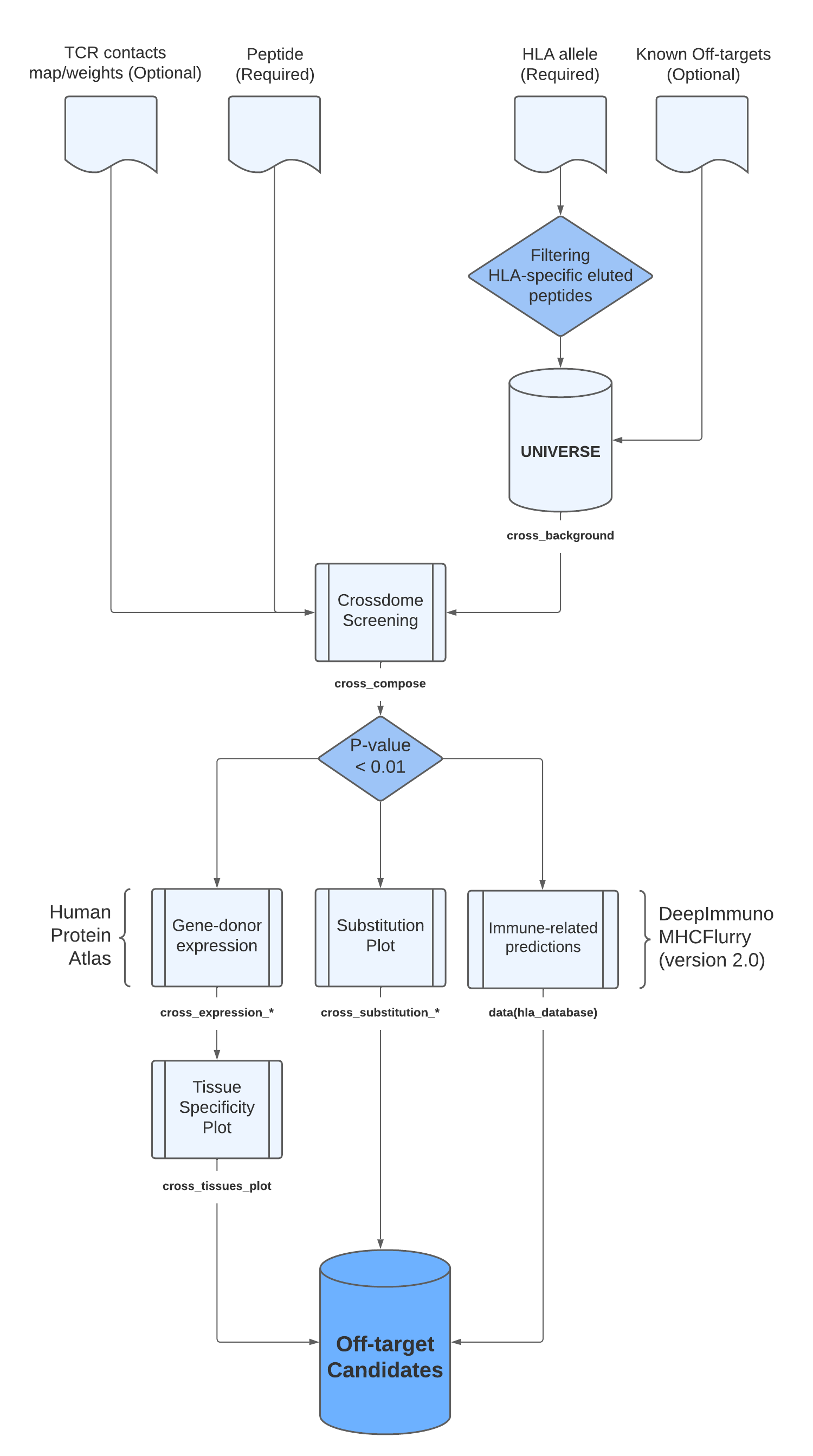

Supplement: Supplementary Figure 4 — CrossDome workflow diagram. The required and optional inputs are displayed on top. Peptide and HLA allele parameters only support 9-mer sequences and Class I alleles, respectively. TCR contacts and known off-targets (e.g., positive controls) can be introduced as optional inputs. The “CrossDome screening” process is performed over a reference “universe” database, using the relatedness score to find biochemically similar peptides. Next, a statistical threshold (p-value < 0.01) and data integration are applied to the potential off-target candidates. The “Gene-donor expression” step maps mRNA expression data from genes to each candidate. The expression profile provides measurements across several healthy tissues. The “Tissue specificity plot” summarizes the expression profile and tissue specificity. Additionally, the “Substitution plot” provides insights into sequence substitution among the best-scored candidates. Finally, the “immune-related predictions” combine immunogenicity and binding affinity predictions for each candidate. The package functions are named below each step in the workflow. [file Image_4.png]
