# Supplementary material for: CrossDome: an interactive R package to predict cross-reactivity risk using immunopeptidomics databases
Source: Front Immunol. 2023 Jun 12;14:1142573. doi: 10.3389/fimmu.2023.1142573 (PMC10291144; doi:10.3389/fimmu.2023.1142573)
Supplement: Supplementary file 8 [file DataSheet_1.pdf]

Supplementary Material

1. **GitHub repository:** The CrossDome tool for T-cell off-target toxicity prediction is under active development, and a beta version is available at the GitHub repository of the Antunes Lab (https://github.com/AntunesLab/crossdome). This repository includes Installation details, summary of CrossDome workflow and strategy, list of functions and features, basic usage, and documentation. The documentation includes vignettes for the package, including basic usage (biochemical profile) and predictions based on the contact map. These vignettes can be accessed through the built-in function "browseVignettes("crossdome")". Further instructions are described on the GitHub README. Note that the repository also includes the reference background dataset used by CrossDome (e.g., hla_database, hpa_database, and peptide_annotation), in addition to the compiled dataset of off-targets used in validation experiments described in the manuscript (e.g., mage_off_targets).

2. **Supplementary Tables:**
    a. **Supplementary Table S1.** Additional information on the 16 validated off-target toxicity cases, involving 4 well-known tumor-associated antigens.
    b. **Supplementary Table S2.** Overlap between sCRAP and the top 50 ranking by CrossDome, for 4 different queries.
    c. **Supplementary Table S3.** Full list of peptides among the top 50 ranking for both sCRAP and CrossDome, for 4 different queries.

3. **Supplementary Figures:**
    a. **Supplementary Figure S1.** Background and validation datasets.
    b. **Supplementary Figure S2.** Dimensionality reduction of biochemical properties.
    c. **Supplementary Figure S3.** Histograms of Coulombic interactions.
    d. **Supplementary Figure S4.** CrossDome workflow diagram.
